# Microcomputed Tomography Studies of the Effectiveness of XP-endo Shaper in Root Canal Preparation: A Review of the Literature

**DOI:** 10.1155/2019/3570870

**Published:** 2019-08-19

**Authors:** Christianne Velozo, Diana Albuquerque

**Affiliations:** Department of Restorative Dentistry and Endodontics, Dental College of Pernambuco, University of Pernambuco, Camaragibe, Brazil

## Abstract

The aim of this study was to undertake a literature review on the use of XP-endo Shaper (FKG Dentaire SA, La Chaux-de-Fonds, Switzerland) for root canal preparation evaluated using microcomputed tomography (micro-CT) technology, with emphasis on the analysis of untouched canal walls. We searched the PubMed, Science Direct, SciELO, and MEDLINE databases for articles published from 2017 to 2019 using the following keywords: micro-CT, untouched walls, XP shaper, and XP endo. Articles without an abstract were excluded. Five papers were selected. Few studies on this topic are available. The studies published so far show that the XP-endo® Shaper system performs well in terms of canal preparation but leaves untouched walls.

## 1. Introduction

The ideal chemical-mechanical preparation should be capable of uniformly flattening the entire perimeter of the root canal and of completely removing the inner layers of contaminated dentin so that the irrigant can reach the full length of the canal space. Such preparation thus allows the removal of persistent bacterial populations, which are an important risk factor for the persistence of periapical disease after endodontic treatment completion, reducing them to levels that enable periradicular tissue healing [1–4].

Within this context, manufacturers have developed projects and placed on the market a myriad of endodontic instruments, including the XP-endo® Shaper instrument introduced in 2015 by FKG Dentaire (La Chaux-de-Fonds, Switzerland). As its name indicates, XP-endo Shaper is an instrument designed for root canal shaping. It performs an asymmetric rotary motion by taking on a semicircular shape when it expands at a temperatures of 35°C or higher. In the early stages of preparation, still at room temperature, the instrument is in the martensitic phase; when introduced into the root canal, its shape changes due to the molecular memory of the austenitic phase. The XP-endo® Shaper has a booster tip that confers a unique geometry, with six cutting edges at the tip and an ISO 15 initial diameter, which gradually increases to a diameter of 30 and 0.01 taper. According to the manufacturer, after expansion, the XP-endo® Shaper reaches a final canal preparation corresponding to a diameter of 30 and 0.04 taper. However, few studies using this system have been conducted. Fabricated with MaxWire alloy (Martensite-Austenite Electropolishing-Flex, FKG), the instrument projects against the walls of the root canal when rotated in asymmetric rotary motion. Thus, XP-endo® Shaper is able to adapt to the morphology of the root canal system, expanding or contracting as it advances along the working length [5, 6].

Various methods have been developed to evaluate the ability of NiTi systems to shape root canals; however, microcomputed tomography (micro-CT), a nondestructive high-resolution three-dimensional X-ray-based imaging technique, is considered the gold standard [7–9]. The aim of this literature review was to survey studies that use micro-CT to evaluate the performance of the XP-endo® Shaper instrument in the preparation of root canals.

## 2. Review

This was a literature review in which the PubMed, Science Direct, SciELO, and MEDLINE databases were searched for articles published from 2017 to 2019. Micro-CT studies published in English that analysed untouched walls after preparation with XP-endo® Shaper were included. The following keywords were used: “XP Endo,” “XP Endo Shaper,” and “micro-CT.” Based on this inclusion criterion, five articles were selected for reading of the full text.

Azim et al. [10] evaluated and compared the shaping ability of XP-endo® Shaper and Vortex Blue using micro-CT. Twenty mandibular incisors with single, long oval-shaped canals (*n* = 10 per group) were scanned before and after crown-down preparation of the root canal. Parameters such as the percentage of untouched walls, canal volume, surface area, amount of dentin removed, debris remaining in the canal, final preparation taper, and total6 time were evaluated. The working length was set at 0.5 mm from the apical foramen. For determination of the final preparation size (diameter and taper), the authors used an innovative method to virtually determine the appropriate gutta-percha cone by superimposing a rounded figure resembling gutta-percha in the most rounded portion of the canal on the micro-CT images. The XP-endo® Shaper performed considerably better in all three thirds (apical, middle, and coronal), with few untouched surfaces (*F* = 25.81, *p* < 0.001). In addition, the XP-endo® Shaper removed more dentin than Vortex Blue. This occurred more in the coronal and middle thirds, but not in the apical third. There was a trend towards less accumulation of debris in the XP-endo® Shaper group. The XP-endo® Shaper was significantly faster than Vortex Blue in instrumenting the root canal space. The XP-endo® Shaper significantly increased the total canal volume (*F* = 77.948, *p* < 0.001), surface area (*F* = 5.543, *p*=0.030), and amount of dentin removed (*F* = 10.044, *p*=0.001) and had significantly less untouched walls (38.6%) compared to Vortex Blue (58.8%). There was less debris at all levels of the canal in the XP-endo® Shaper group. The results were almost significant (*p*=0.059). The XP-endo® Shaper was also significantly faster in completing the mechanical preparation of the root canal space by almost 1 minute (*t* = 6.216, *p* < 0.001). The XP-endo® Shaper can prepare and touch more walls in oval canals compared to Vortex Blue. However, the final preparation taper varies according to the anatomy of the treated tooth.

The cleaning and shaping ability of three instrumentation systems (Self-Adjusting File (SAF; ReDentNOVA, Ra'anana, Israel), TRUShape (Dentsply Sirona, Tulsa, OK, USA), and XP-endo® Shaper (FKG Dentaire, La Chaux-de-Fonds, Switzerland)) were evaluated by Lacerda et al. [11] in oval canals. The volume, surface area, and untouched areas were measured. The distal canals of mandibular molars were digitized by micro-CT before and after preparation, in addition to postpreparation histologic analysis. The working length for preparation was set at 1 mm from the apical foramen. There was no significant difference in the amount of unprepared surfaces between the three systems studied for length, canal volume, or surface area of the canal before preparation (*p* > 0.05), except for the comparison in the apical 4 mm segments between SAF and XP-endo® Shaper. The mean percentage of untouched areas was 9.85%, 15.88%, and 17.77% for the SAF, TRUShape, and XP-endo® Shaper systems, respectively. The mean number of untouched areas after preparation with SAF, TRUShape, and XP-endo® Shaper was 10.92%, 17.45%, and 17.31%, respectively. No statistically significant differences were observed between the three systems (*p* > 0.05). None of the systems prepared 100% of the root canal walls. The cleaning ability of the three systems was similar. TRUShape and XP-endo® Shaper are recently launched instruments, and studies evaluating their cleaning and shaping ability are scarce.

Versiani et al. [12] investigated the shaping ability of the XP-endo® Shaper (FKG Dentaire SA, La Chaux-de-Fonds, Switzerland), iRaCe (FKG Dentaire SA), and EdgeFile (Edge Endo, Albuquerque, NM, USA) systems using micro-CT. The recorded images obtained before and after preparation were evaluated regarding 3D (length, volume, surface area, structure model index (SMI), and untouched areas) and 2D morphometric parameters (area, perimeter, roundness, and major and minor diameter). For the 3D and 2D parameters, the long oval-shaped canal was classified based on major and minor diameter and the samples were matched to increase the internal validity of the study. The sample size was *n* = 10 per group. The working length was established at 0.5 mm from the apical foramen. The XP-endo® Shaper, iRaCe, and EdgeFile systems exhibited a similar shaping ability. No significant difference in the amount of untouched canal walls was observed between groups. The XP-endo® Shaper system significantly changed the overall geometry of the root canal to a more conical shape (SMI = 2.59) when compared to the iRaCe (SMI = 2.34) and EdgeFile (SMI = 2.28) systems (*p* < 0.05). There was no significant difference between groups for the mean percent increase in volume (∼52%) and surface area (10.8%–14.2%) or mean percentage of the untouched surface area (9.42%) (*p* > 0.05). None of the techniques was able to completely prepare the long oval-shaped canals of mandibular incisors.

The shaping ability of the XP-endo® Shaper instrument (FKG Dentaire SA, La Chaux-de-Fonds, Switzerland) was evaluated in the study by De-Deus et al. [13]. Mesial roots of mandibular molars (*n* = 10) were scanned by micro-CT and prepared with the XP-endo® Shaper instrument up to a working length of 1 mm from the apical foramen according to the manufacturer's recommendation. Each specimen was then submitted to an extra 15, 30, and 45 s of active instrumentation at the same length. The pre- and postpreparation datasets recorded by micro-CT were examined for the percentage of volume and surface area of the instrumented canal, surface area of the noninstrumented canal, and volume of removed dentin. In that study, the extra activation time of the XP-endo® Shaper instrument at working length resulted in a comprehensive root canal preparation, increasing the volume and surface area of the root canal and the volume of removed dentin. The XP-endo® Shaper provided a preparation that ranged from 31.82% of the surface area of untouched canal walls when the activation time recommended by the manufacturer was used at working length to 29.27% for the extra time of 15 s, 25.0% for the extra time of 30 s, and 22.74% when an extra time of 45 s was used at the same working length. Nevertheless, this instrument could not completely incorporate all canal walls during preparation.

In a micro-CT study, Zhao et al. [14] evaluated the Reciproc Blue and XP-endo Shaper systems regarding the area of untouched canal walls (AUCW), accumulated hard tissue debris (AHTD), and the efficacy of three irrigation protocols on the percent reduction of debris in C-shaped canals of mandibular molars. Seventy mandibular molars with C-shaped canals were scanned and allocated to two shaping groups (*n* = 35): Reciproc Blue and XP-endo Shaper. After instrumentation, the specimens were assigned to three irrigation subgroups (*n* = 10): syringe and needle irrigation (SNI), XP-endo Finisher (XP-F; FKG Dentaire), and passive ultrasonic irrigation (PUI). The AUCW and AHTD after instrumentation and percent reduction of AHTD after irrigation were calculated from the micro-CT scans. The data were analysed by comparisons of two groups (Reciproc Blue and XP-endo Shaper) or multiple subgroups followed by pairwise comparison procedures (SNI, XP-F, and PUI) at *α* = 0.05. For Reciproc Blue and XP-endo Shaper, 33.04% and 30.45%, respectively, of the canal wall remained untouched (*p* > 0.05). In both groups, the apical third had greater AUCW than the coronal third (*p* < 0.05). The Reciproc Blue and XP-endo Shaper systems were associated with similar AUCW after instrumentation of C-shaped canals. Reciproc Blue left significantly higher levels of AHTD compared to XP-endo Shaper. PUI and XP-F irrigation removed more debris than SNI when the Reciproc Blue system was used.

This review evaluated the effectiveness of XP-endo® Shaper, preparing geometrically the root canal system using micro-CT technology. The results found in the few articles published so far demonstrate different percentages of untouched walls (Table 1). These differences can be explained by methodological variations among studies. According to Azim et al. [10], 38.6% of the prepared canal walls of mandibular incisors remained untouched. However, in the same year, Lacerda et al. [11], preparing distal roots of mandibular molars, obtained 17.31% of untouched walls. Versiani et al. [12], preparing mandibular incisors with this instrument, reported 9.42% of untouched walls. Recently, De-Deus et al. [13] found 31.82% of untouched walls in the preparation of mesial roots, while Zhao et al. [14] reported a percentage of 30.45% in the same year. These values were obtained for preparations performed according to the manufacturer's recommendation.

The studies by Azim et al. [10], Versiani et al. [12], and Zhao et al. [14] established anatomical correspondences of the samples and determined the morphology of root canals in order to increase the internal validity of the analyses. However, despite the rigour of sample selection, the percentage of untouched walls obtained after preparation with the XP-endo® Shaper system ranged from 38.6% to 9.42%. Azim et al. [10] and Versiani et al. [12] used the classification of long oval-shaped canals [15] for mandibular incisors. The sample used by Versiani et al. [12] was selected based on micro-CT measurements, which is the gold standard for anatomical assessments [7–9], while in the study of Azim et al. [10], the samples were selected using periapical radiographs and cone beam CT. Zhao et al. [14] also selected the samples based on micro-CT measurements and obtained a significantly higher percentage than Versiani et al. [12] using the same technology. However, it is not possible to compare these studies because of the morphological difference in the root canals of the samples. Zhao et al. [14] used C-shaped (irregular) canals of mandibular second molars. It is important to stress that the micro-CT analysis of Versiani et al. [12] considered the morphological parameters of major and minor diameter of the surface area of root canals, and Zhao et al. [14], for combination of the samples, used 3D morphology, length, preoperative volume, and surface area of the root canals. Establishing a set of parameters for sample selection in micro-CT studies may favour comparison between these studies given that the morphologies are compatible. The wide variation in the percentage of untouched walls in micro-CT studies might be related to differences in tooth morphology and in the characteristics of the instruments used, as well as methodological differences [16–19]. In the study by Azim et al. [10], the working length was set at 0.5 mm from the apical foramen. The authors observed that, at this length, 40% of the instrumented samples in the XP-endo® Shaper group showed areas of packed debris or no alteratison in canal volume, indicating that the file did not reach the level of 0.5 mm. According to the authors, these results suggest that XP-endo® Shaper may only expand in the mesiodistal and buccolingual direction but not in the coronoapical direction. In the study by Versiani et al. [12], the working length was also set at 0.5 mm from the apical foramen. At this length, the authors found a mean taper of 0.02 before preparation and of 0.06 after preparation in the mesiodistal direction of the canal. However, in the buccolingual direction, the mean taper decreased from 0.10 to 0.08 after preparation, suggesting the accumulation of debris. Zhao et al. [14], De-Deus et al. [13], and Lacerda et al. [11] used a working length of 1 mm from the apical foramen. However, comparison of the results was not possible because of the samples selected for these studies: C-shaped mandibular molars, mesial roots of mandibular molars, and distal roots of mandibular molars, respectively. These anatomical variations impaired comparisons of the results obtained in these studies.

Regarding the distribution of the samples, the experimental groups consisted of *n* = 10 in the studies by De-Deus et al. [13], Versiani et al. [12], and Azim et al. [10], while Lacerda et al. [11] used *n* = 11 and Zhao et al. [14] used *n* = 35. The last authors used a larger *n* because the samples were subdivided for the evaluation of irrigation methods.

The SMI parameter is commonly used to analyse to changes in the dimensional shape of the root canal, which evaluates surface convexity. Studies have shown that SMI values increased after preparation, indicating that the irregular, small, and flat conical canal changed to a more rounded and smoother conical canal [16–22]. Among the studies using micro-CT analysis and XP-endo® Shaper, only the study by Versiani et al. [12] analysed this parameter and reported an SMI of 2.59 for this instrument.

## 3. Conclusion

The studies published so far show that XP-endo® Shaper exhibits good performance in root canal preparation but leaves untouched walls. Despite the rigour of sample selection using micro-CT technology, comparison of the results of untouched area analysis requires compatible anatomies samples for the use of any endodontic instrument.

## Figures and Tables

**Table 1:** Synthesis of the included articles on XP-endo® shaper evaluated by micro-CT.

Author and year	Teeth	Method	Result
Azim et al. [10]	20 mandibular incisors	Evaluation of root canal preparation with the XP-endo Shaper and Vortex Blue instruments by micro-CT (*n* = 10)	XP-endo® Shaper had significantly less untouched walls (38.6%) compared to Vortex Blue (58.8%). However, the final preparation taper varied according to the anatomy of the treated tooth.
Lacerda et al. [11]	33 distal canals of mandibular molars	Evaluation of root canal preparation with the XP-endo Shaper, SAF, and TRUShape instruments by micro-CT and histologic analysis (*n* = 11)	There was no significant difference in the amount of unprepared surfaces between the three systems studied. The mean number of untouched areas after preparation with SAF, TRUShape, and XP-endo® Shaper was 10.92%, 17.45%, and 17.31%, respectively. Except for comparison in the apical 4 mm segments between SAF and XP-endo® Shaper, the latter showed better performance.
Versiani et al. [12]	30 mandibular incisors	Evaluation of root canal preparation with the XP-endo Shaper, iRace (R1, R2, and R3), and EdgeFile (X1 and X7) instruments by micro-CT (*n* = 10)	The XP-endo® Shaper, iRace, and EdgeFile system showed a similar shaping ability. The mean percentage of the untouched surface area was 9.42% (*p* > 0.05) for XP-endo® Shaper. There was no significant difference in the amount of untouched canal walls between groups.
De-Deus et al. [13]	10 mesial canals of mandibular molars	Micro-CT analysis of the shaping ability of the XP-endo Shaper instrument as recommended by the manufacturer and then submitting each specimen to an extra activation time of 15, 30, and 45 s (*n* = 10)	The extra activation times of XP-endo Shaper at working length resulted in a comprehensive root canal preparation, increasing volume and surface area of the root canal and volume of removed dentin. Preparation ranging from 31.82% of untouched surface area using the time recommended by the manufacturer at working length to 29.27%, 25.0%, and 22.74% when an extra time of 15, 30, and 45 s was used at the same length, respectively.
Zhao et al. [14]	75 mandibular molars	Evaluation of root canal preparation with the Reciproc Blue and XP-endo Shaper instruments by micro-CT (*n* = 35)	The XP-endo Shaper and Reciproc Blue systems were associated with a similar area of untouched canal walls after instrumentation of C-shaped root canals, in which 30.45% and 33.04% of canal walls remained untouched, respectively (*p* > 0.05).
